# The Tomato *Hoffman’s Anthocyaninless* Gene Encodes a bHLH Transcription Factor Involved in Anthocyanin Biosynthesis That Is Developmentally Regulated and Induced by Low Temperatures

**DOI:** 10.1371/journal.pone.0151067

**Published:** 2016-03-04

**Authors:** Zhengkun Qiu, Xiaoxuan Wang, Jianchang Gao, Yanmei Guo, Zejun Huang, Yongchen Du

**Affiliations:** The Institute of Vegetables and Flowers, Chinese Academy of Agricultural Sciences, Beijing, People’s Republic of China; National Taiwan University, TAIWAN

## Abstract

Anthocyanin pigments play many roles in plants, including providing protection against biotic and abiotic stresses. Many of the genes that mediate anthocyanin accumulation have been identified through studies of flowers and fruits; however, the mechanisms of genes involved in anthocyanin regulation in seedlings under low-temperature stimulus are less well understood. Genetic characterization of a tomato inbred line, FMTT271, which showed no anthocyanin pigmentation, revealed a mutation in a bHLH transcription factor (TF) gene, which corresponds to the *ah* (*Hoffman's anthocyaninless*) locus, and so the gene in FMTT271 at that locus was named *ah*. Overexpression of the wild type allele of *AH* in FMTT271 resulted in greater anthocyanin accumulation and increased expression of several genes in the anthocyanin biosynthetic pathway. The expression of *AH* and anthocyanin accumulation in seedlings was shown to be developmentally regulated and induced by low-temperature stress. Additionally, transcriptome analyses of hypocotyls and leaves from the near-isogenic lines seedlings revealed that *AH* not only influences the expression of anthocyanin biosynthetic genes, but also genes associated with responses to abiotic stress. Furthermore, the *ah* mutation was shown to cause accumulation of reactive oxidative species and the constitutive activation of defense responses under cold conditions. These results suggest that *AH* regulates anthocyanin biosynthesis, thereby playing a protective role, and that this function is particularly important in young seedlings that are particularly vulnerable to abiotic stresses.

## Introduction

Anthocyanins, the plant pigments responsible for red, purple and blue colors in flowers and fruits, act as visual cues to attract insects that pollinate and help disperse seeds [1]. They are also synthesized in vegetative tissues and have been referred to as ‘Nature’s Swiss Army Knife’ due to their diverse roles in protecting against biotic stress and abiotic stresses, including those caused by insects, phytopathogens, drought, UV irradiation and low temperatures [2–5]. In addition, anthocyanins were recently shown to delay over-ripening in tomato (*Solanum lycopersicum*) fruits and their enhanced accumulation resulted in a major increase in fruit shelf-life [6, 7]

The anthocyanin biosynthetic pathway is one of the most well studied secondary metabolite pathways and it has been shown to be highly conserved across plant taxa [8, 9]. Many of the genes that encode enzymes involved in anthocyanin biosynthesis have been well characterized, and they can be divided into two groups: the early biosynthetic genes (EBGs; including *CHS*, encoding chalcone synthase; *CHI*, chalcone isomerase; and *F3H*, flavanone 3-hydroxylase) that are common to different flavonoid sub-pathways, and the late biosynthetic genes (LBGs; *F3’5’H*, encoding flavonoid 3’5’-hydroxylase; *DFR*, dihydroflavonol 4-reductase; *ANS*, anthocyanidin synthase; *3GT*, flavonoid 3-O-glucosyltransferase; *RT*, anthocyanin rhamnosyltransferase; *AAC*, anthocyanin acyltransferase; *5GT*, flavonoid 5-O-glucosyltransferasese; *GST*, glutathione *S*-transferase), which contribute to anthocyanin and proanthocyanidin biosynthesis [10, 11]. Anthocyanin biosynthesis is regulated by the combined action of R2R3-MYB and bHLH transcription factors (TFs), as well as WD40-repeat proteins [9]. Many of these TFs have been identified in several model species, including maize (*Zea mays*), petunia (*Petunia×hybrida*) and *Arabidopsis thaliana* [11–18].

Basic helix-loop-helix (bHLH) TFs are well known to play key roles in the regulation of anthocyanin biosynthesis in plants [9]. The maize *Lc* gene was the first plant bHLH TF to be identified [16], and overexpression of *Lc* has been shown to significantly increase the anthocyanin content in several species [10, 13, 19, 20]. The bHLH genes *R*, *B*, *Sn* and *Hopi* were subsequently identified in maize and shown to induce tissue-specific anthocyanin biosynthesis, including expression in the aleurone layer, scutellum, pericarp, root, mesocotyl, leaf and anther [15, 18, 21, 22]. AN1, a bHLH protein that interacts with R2R3-MYB AN2 and WD40 AN11, was shown to specifically control anthocyanin accumulation in petunia petals and anthers [17, 23, 24], and another petunia bHLH protein, JAF13, interacts with AN2, thereby activating anthocyanin biosynthetic genes [23]. *JAF13* does not appear to be functionally equivalent to *AN1*, since its expression does not complement the *an1* mutant [25]. In *A*. *thaliana*, there are 133 bHLH genes, of which three have been confirmed to be related to anthocyanin formation [26]. The best studied is *TT8*, which is a key regulator of anthocyanin and proanthocyanidin (PA) biosynthesis [11]. It has been shown that TT8 can regulates its own expression through a positive feedback loop [27, 28]. The other two are *GL3* and *EGL3*, which act in vegetative tissues [29, 30].

Among abiotic environmental stresses, low temperature affect plant growth most seriously [31]. Plants respond to low temperature with a number of metabolism changes, of which one is modulating the anthocyanin content. Previous studies have shown that low temperatures stimulate anthocyanin accumulation by upregulating the expression of anthocyanin biosynthetic genes [32–34]. Several R2R3-MYB TFs, including *BoPAP1*, *NtAN2* and *SlAN2*, have recently been shown to control anthocyanin production under low temperature conditions [35–37]; however, only a few cases of bHLH TFs being involved in the regulation of anthocyanin biosynthesis under low temperature stress have been reported.

Tomato is one of the most widely consumed vegetables in the world, and improvement of its resistance to abiotic stresses is central to many tomato breeding programs. To date, more than 20 tomato mutants with altered anthocyanin biosynthesis have been reported, but the underlying genes of only three have been identified by map-based cloning: *anthocyanin without* (*aw*), *anthocyanin free* (*af*) and *anthocyanin reduced* (*are*), which encode a dihydroflavonol 4-reductase (DFR), a chalcone isomerase (CHI) and a flavonoid 3-hydroxylase (F3H), respectively [38–41]. T-DNA activation-tagging experiments in tomato lead to the identification of an R2R3-MYB TF, *anthocyanin 1* (*ANT1*), which shares high homology with petunia *AN2* [42], and which was shown to control anthocyanin accumulation. Finally, three R2R3-MYB TFs, *SlAN2*, *SlAN2-like* and *SlANT1-like*, were also reported to control anthocyanin accumulation in tomato [36, 43]. Overexpression of the MYB TFs related to anthocyanin biosynthesis in tomato enhanced the anthocyanin content in leaves and fruits to varying degrees [42–44]. Most of the identified genes related to anthocyanin biosynthesis to date belong to the R2R3-MYB TF family; however, some bHLH TFs have also been shown to be involved in this process in tomato.

In this paper, we describe the characterization of a tomato genotype FMTT271, developed by conventional breeding approaches, which produces no anthocyanin in hypocotyls, leaves, buds and flowers at any developmental stage. The defective gene responsible for this abnormal phenotype was identified by map-based cloning and shown to be a bHLH TF gene, which we named *AH*. Expression analysis and RNA sequencing (RNA-seq)-based transcriptome analysis demonstrated that *AH* serves as a master regulator of anthocyanin biosynthesis in tomato.

## Materials and Methods

### Plant material and growth conditions

*S*. *lycopersicum* FMTT271 was developed by conventional breeding procedures in the Institute of Vegetables and Flowers, CAAS (Beijing, China). Seeds of *S*. *pennellii* LA716 and *S*. *lycopersicum* LA0260 were obtained from the Tomato Genetics Resource Center (http://tgrc.ucdavis.edu/). Using molecular marker-assisted technology, a series of advanced backcrossed lines and near-isogenic lines (NILs) were developed using the wild-type tomato LA716 as a donor parent and FMTT271 as the recipient parent. The seedlings used for the mapping experiments were grown in 32-plug trays containing sterilized soil in a growth chamber under 16-h day and 8-h night conditions. At the eight-leaf stage, the seedlings were transplanted to a greenhouse at the farm of the Institute of Vegetables and Flowers, CAAS (Beijing, China). For developmental analyses, sterilized seeds of NIL-PH and NIL-GH plants were placed in 250 mL flasks containing 80 mL half Hoagland nutrition solution/0.7% agar [45]. Mixed hypocotyls from 20 lines were used for RNA extraction and qPCR analyses. For temperature treatments, five-leaf-old seedlings were cultivated in a phytotron under either 16 h of light at 28°C/ 8 h of dark at 20°C, or 16 h of light at 16°C/ 8 h of dark at 8°C. Leaves from single lines were used for anthocyanin extraction and quantification, RNA extraction and subsequent qPCR analyses. Three biological replicates of all samples were performed.

### Anthocyanin extraction and quantification

Anthocyanin extraction and quantification was performed as previously described [46]. Briefly, 1 g fresh weight (FW) hypocotyl or leaf material was transferred into a tube containing 4.3 mL of extraction solution (1-propanol/HCl/distilled water, 18/1/81, v/v/v). The tubes were then placed in boiling water for 6 min and incubated in the dark overnight at room temperature. An additional 3.7 mL of extraction solution was then added to the mixture, the sample was mixed and centrifuged at 1,000 g for 5 min. The supernatant was filtered through a 0.45 μm filter (Millipore), and the amount of anthocyanin in the extracts was quantified using a spectrophotometer by reading at A535 and A650 and expressed as (A535-A650) per gram of FW. Each analysis was performed with three biological replicates.

### Mapping and cloning of *ah*

The *ah* locus was mapped to an interval between CAPS markers C2_At2g32600 and C2_At2g47580 on chromosome 9 using 12 BC_1_ plants and the molecular markers from TOMATO-EXPEN 2000 [47]. The *ah* locus was then further narrowed down to a 130-kb genomic region between the CAPS markers CAPS2 and CAPS4 using 1,458 BC_4_F_1_ plants and additional molecular markers. The primers used for mapping are listed in S7 Table. The open reading frame (ORF) of the candidate gene, *Solyc09g065100*, was amplified from genomic DNA from both the wild-type and the *ah* mutant using primers lqF (5’-ATGGAGATTATACAGCCTAATAG-3’) and lqR (5’-TTAATTAACTCTAGGGATTATC-3’). The PCR products were then sequenced.

### Sequence alignment and phylogenetic analysis

**Fig 1:**
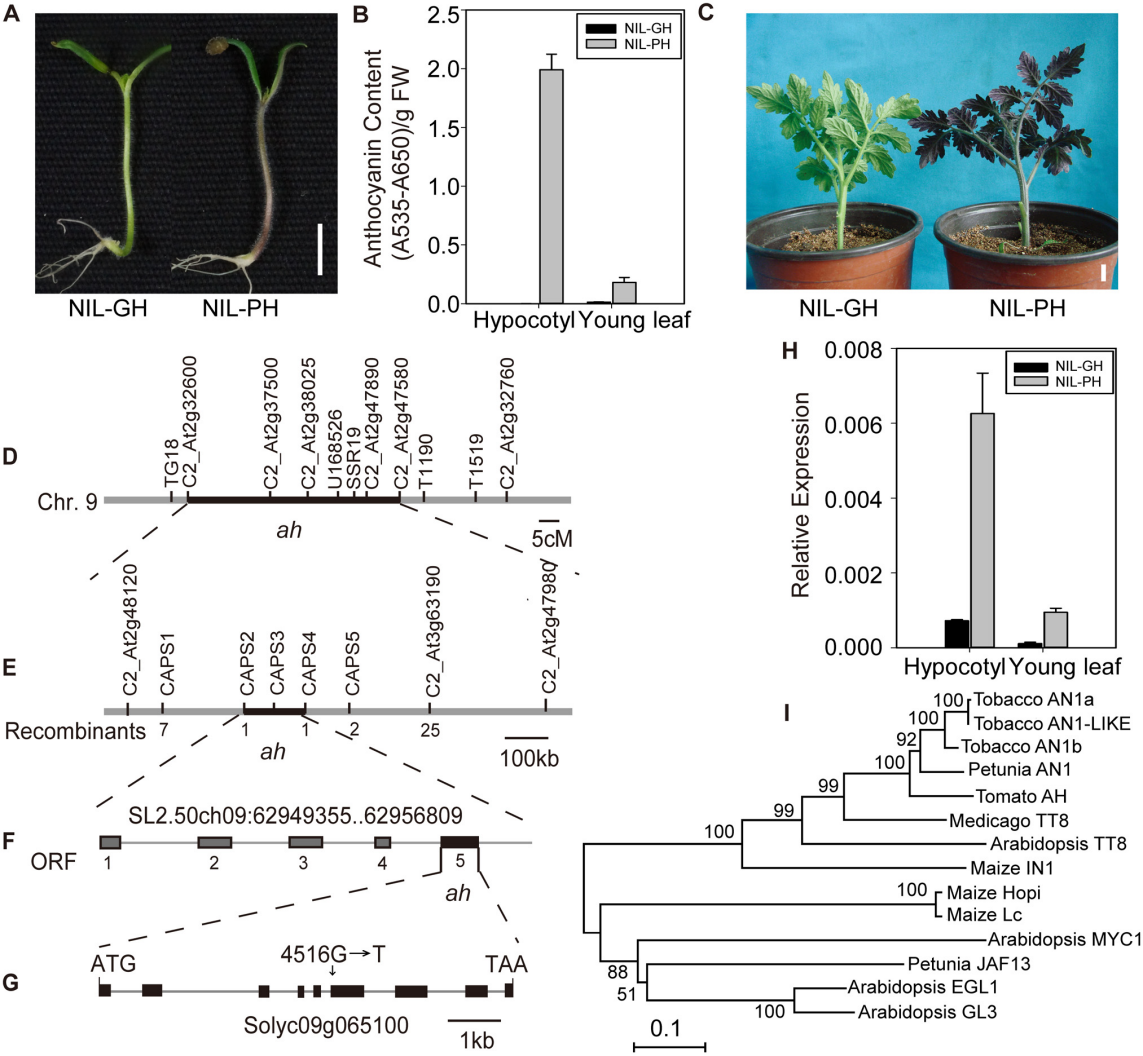
Map-based cloning of the *ah* locus. (A) Phenotypes of the NIL seedlings. (B) Total anthocyanin content in hypocotyls and leaves of NIL-PH and NIL-GH plants. (C) The phenotype of 5-leaf-old NIL-PH and NIL-GH plants after growth in a phytotron under 16 h of light at 16°C/ 8 h of dark at 8°C for 20 days. (D) Coarse linkage map of the green locus on chromosome 9, and high-resolution linkage map of *ah* (E), with the number of recombinants between the molecular marker and *ah* indicated. (F) Annotation of the candidate region surrounding *ah*, with dark gray boxes indicating the putative genes predicted in ITAG2.40. (G) *AH* structure and the mutation site in FMTT271. The black boxes represent the coding sequences and lines between boxes represent introns. (H) Relative expression levels of *AH* in hypocotyls and leaves of NIL-PH and NIL-GH plants. A tomato *ACTIN* (*Solyc03g078400*) gene was used as the reference gene. The hypocotyls from 6-old day and the leaves from 5-leaf-day seedlings were used. (I) Phylogenetic tree of AH and other bHLH proteins from several plant species, constructed using the neighbor-joining method. The bHLH proteins and their respective GenBank accession numbers are as follows: petunia AN1, AAG25927; petunia JAF13, AAC39455; maize Lc, NP_001105339; *Arabidopsis* MYC1, NP_191957; tobacco AN1b, AEE99258; tobacco AN1a, AEE99257; *Arabidopsis* GL3, NP_680372; *Arabidopsis* EGL3, NP_176552; *Arabidopsis* TT8, CAC14865; maize IN1, AAB03841; *Medicago* TT8, XP_003590656; tobacco AN1-LIKE, NP_001289495; maize Hopi, CAB92300. NIL-PH (GH) refers to the plants from the NIL population with purple (green) in hypocotyls. Three biological replicates of all samples were analyzed. Scale bars, 1 cm.

The deduced amino acid sequences of AH and 13 other bHLH protein sequences, obtained from GenBank (http://www.ncbi.nlm.nih.gov/genbank/), were aligned using the MEGA v 5.05 and Clustal W software (for accession numbers see Fig 1 legend) [48]. Alignment parameters (gap opening penalty and gap extension penalty) used were 10.00 and 0.1 for pair-wise alignments, and 15.00 and 0.30 for multiple alignments. A phylogenetic tree was constructed and visualized using the neighbor-joining (NJ) method in MEGA v 5.05. The statistical significance of individual nodes was assessed by bootstrap analyses with 1,000 replicates.

### Plasmid construction and plant transformation

Total RNA was extracted from the 2-day old hypocotyls of *S*. *pennellii* LA716 using the PureLink RNA Mini Kit (Invitrogen) according to the manufacturer’s instructions. Reverse transcription (RT) was performed using random primers, an oligo (dT) 15 primer and the GoScript RT System (Promega). The full-length *AH* ORF was identified based on cDNA sequence that was amplified by PCR using the primers (*AH*F, 5’- CCATTCTAGAATGGAGATTATACAGCCTAATAG -3’, and *AH*R, 5’- CTATCCCGGGTTAATTAACTCTAGGGATTATC -3’), which had *Xba*I and *Sma*I recognition sites, respectively. The PCR product was inserted into the binary vector pBI121 (Clontech) downstream of the cauliflower mosaic virus 35S promoter sequence. The sequence of the resulting *pBI121-AH* plasmid was verified by sequencing and introduced into the *ah* mutant FMTT271 by *Agrobacterium tumefaciens* (GV3101)-mediated transformation, as previously described (Park et al. 2003). Transgenic plants were confirmed by PCR using the NTP II-specific primers (NTPIIF, 5’-AGACAATCGGCTGCTCTGAT-3’, and NPTIIR, 5’-TCATTTCGAACCCCAGAGTC-3’).

### Gene expression analyses by qPCR

Total RNA was isolated and purified as described above. Reverse transcription (RT) was performed using random primers and an oligo (dT) 15 primer and the GoScript RT System (Promega). Quantitative real-time PCR was performed using a LightCycler 480 SYBR Green I Mastermix (Roche) on a LightCycler 480 real-time PCR instrument (Roche). The 2^-ΔΔ^Ct method was used to calculate the relative expression of each gene [49]. Primer pairs are listed in S7 Table. A tomato *ACTIN* (*Solyc03g078400*) gene was used as the reference gene and all analyses were performed using three technical and three biological replicates.

### RNA transcriptome analyses using RNA-seq

For hypocotyl analysis, total RNA was extracted from the hypocotyls of 20 5-day old seedlings cultivated in flasks containing half Hoagland nutrition solution, as described above. For temperature assays, total RNA was also extracted from the leaves of single 5-leaf old seedlings cultivated in a phytotron for 5 days under either 16 h of light at 28°C/ 8 h of dark at 20°C, or 16 h of light at 16°C/ 8 h of dark at 8°C. Two biological replicates were performed for RNA transcriptome analyses. RNA-seq analysis was carried using an Illumina Hiseq2000 (Berry Genomics Company). The cleaned reads were aligned to the tomato genome sequence SL2.50 (Sol Genomics Network) using the Tophat software [50, 51], allowing one mismatch. The resulting alignments were assembled using Cufflinks in order to generate unique sequences using the ITAG2.4 gene model (Sol Genomics Network). The statistical package DEGseq was used to calculate p values with the MA-plot-based method [52]. Fold changes (log_2_ ratio) were calculated on the basis of RPKM values. A log2 ratio ˃ 1 or < -1 and P ˂ 0.01 were considered to be the threshold for identification of differentially expressed genes (DEGs). Gene ontology (GO) analysis of DEGs was performed using DAVID (The Database for Annotation, Visualization and Integrated Discovery, https://david.ncifcrf.gov/) with the most homologous *A*. *thaliana* genes.

### Histochemical staining assay

3,3’-diaminobenzidine (DAB) staining and trypan blue staining were performed as previously described [53, 54] on 5-leaf old seedlings cultivated in a phytotron for 3 days under 16 h of light / 8 h of dark at 4°C. The leaves from individual lines were used for DAB staining and trypan blue staining. Three biological replicates were performed.

## Results

### The FMTT271 inbred tomato line displays an anthocyanin-deficient phenotype

Tomato seedlings typically have purple pigmentation on their hypocotyls when grown under normal growth conditions; however, the plants of the FMTT271 inbred tomato line were green (Fig 1A). To elucidate the genetic basis of this phenotype, a series of advanced backcrossed lines and a NIL population were developed using the wild-type tomato genotype *S*. *pennellii* LA716 as the donor parent and FMTT271 as the recipient parent. Compared with the purple hypocotyl plants (NIL-PH) from the NIL population, the green hypocotyl plants (NIL-GH) lacked visible anthocyanin pigments in all tissues/organs, including hypocotyls, leaves, buds, sepals and the center axis of the petals (Fig 1A and S1 Fig). Analysis of extracts from the hypocotyls of 5-day old purple (PH) and green (GH) seedlings from the NIL population indicated an absence, or barely perceptible levels, of anthocyanins in the GH samples, while they were clearly detected in the PH samples (Fig 1B). Anthocyanins were also visibly detectable in extracts from young leaves of 5-leaf old purple (NIL-PH) tomato; however, the total anthocyanin levels in leaves were much lower than those in hypocotyls (Fig 1B). In addition, even when grown under low temperature conditions (16°C/8°C, day/night, 20 days), a stress known to promote anthocyanin biosynthesis in plants [32–34], the NIL-GH did not accumulate anthocyanins in any part of the plant, while the whole plants of NIL-PH turned dark purple (Fig 1C).

### Map-based Cloning of the *ah* Locus

We observed that all of the F_1_ plants showed a purple coloration in their hypocotyls. Segregation in the BC_1_ and BC_4_S_1_ backcross (BC) population was consistent with a single locus inheritance (1:1 and 3:1, respectively, purple hypocotyls versus green hypocotyls; S1 Table). These results indicated that the green phenotype of FMTT271 is controlled by a single recessive locus. Using the BC_1_ population of 12 individuals, the locus was mapped to a 44.5 centimorgan (cM) region on the long arm of chromosome 9, between the flanking CAPS markers C2_At2g32600 and C2_At2g47580 (Fig 1D). The *ah* (*Hoffman's anthocyaninless*) locus, resulting in tomato plants completely free of anthocyanins, lies within this region [55]. Therefore, an allelism test was performed to determine whether the green locus was an allele of *ah*. F_1_ hybrids from crosses between FMTT271 and the *ah* mutant LA0260 all showed no anthocyanin pigments (S2A Fig), indicating that the green locus in FMTT271 is an allele of *ah*. Thus, we named this single, recessive gene *ah*. Further fine mapping, using a BC_4_F_1_ mapping population, revealed *ah* to be located in a 130-kb interval between the CAPS markers, CAPS2 and CAPS4 (Fig 1E). A total of 5 candidate genes (ITAG 2.40) were predicted to be present in this region (Fig 1F, S2 Table), and a sequence similarity search predicted that *Solyc09g065100* from this region was similar to the *A*. *thaliana* bHLH transcription factor, *TT8*. Quantitative real time PCR (qPCR) analysis showed that *Solyc09g065100* transcript levels were higher in the hypocotyls than in the leaves of NIL-PH plants (Fig 1H), which corresponded to the relative anthocyanin concentrations in the two organs (Fig 1B). We also found that expression of *Solyc09g065100* was much higher in purple NIL compared to green NIL plants, both in hypocotyls and in leaves (Fig 1H). Taken together, these results suggested that *Solyc09g065100* was the best candidate gene for the *ah* locus.

Sequencing of the predicted full-length cDNA of *Solyc09g065100*, amplified by reverse transcription (RT)-PCR of the *AH/AH* and *ah/ah* genotypes, revealed a single G to T substitution at base 550 of the cDNA clone (exon 6 in the genomic clone) in the *ah* mutant (Fig 1G). This substitution is predicted to result in the conversion of glycine (Gly) 184 to a stop codon, truncating the predicted protein by 501 amino acids, and resulting in the loss in the translated protein of a polypeptide region that includes the bHLH domain and the ACT-like domain (S3 Fig). Besides, the mutant LA0260 was found to harbor an identical mutation in *AH* (S2B Fig). However, this mutation was not detected in the wild-type plant, Heinz1706 (S2B Fig). In addition to the sequence of cDNA, 28 single nucleotide polymorphisms (SNPs) and 2 inserts/deletions were founded in the putative promoter (-984 bp relative to the start codon of *AH*) between LA716 and FMTT271 (S4 Fig).

*AH* encodes a putative TF with a bHLH domain. A BLAST search (http://blast.ncbi.nlm.nih.gov/Blast.cgi) using the AH protein sequence revealed that the protein with the highest sequence similarity was a bHLH protein from *N*. *tabacum* (75%), while homologs from petunia (AN1) and *A*. *thaliana* (TT8) (S3 Fig), both of which are required for the regulation of anthocyanin biosynthesis [11, 17], showed 71% and 24% sequence similarity, respectively. Due to the strong homology of AH to petunia AN1, the AH were also named SlAN1 [36]. An amino acid sequence alignment showed that a Myb-interaction region, a bHLH domain and an ACT-like domain are highly conserved among these proteins (S3 Fig). A phylogenetic analysis of the relationship between AH and other bHLH homologs associated with anthocyanin biosynthesis showed that AH, petunia AN1, NtAN1b and TT8 clustered within the same clade (Fig 1I).

**Fig 2:**
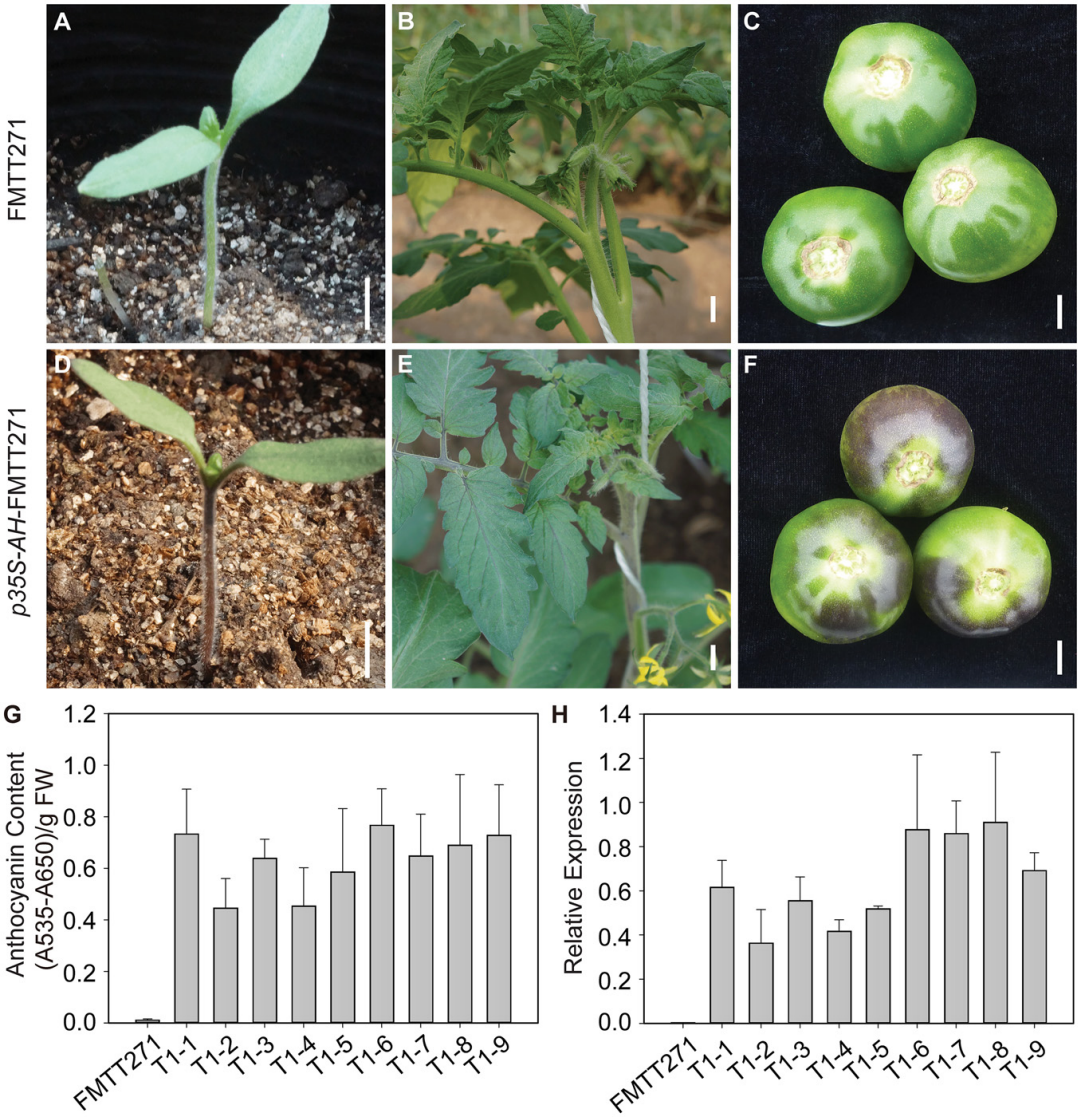
Analyses of *AH* expression and anthocyanin content in leaves of control and *AH* over-expressing transgenic tomato lines. (A-F) Phenotypes of hypocotyls, leaves and fruits of the control and *AH*-expressing lines. Scale bars, 1 cm. (G) Total anthocyanin content in leaves of the control and *AH*-expressing lines (T1-1 to T1-9). (H) Relative expression levels of *AH* in leaves of the control and *AH*-expressing lines (T1-1 to T1-9). T1 generation plants were used for the analyses. Data presented here are the means of three replicates with error bars indicating ±SD.

To confirm that *AH* regulates anthocyanin biosynthesis in tomato, the full-length *AH* cDNA was expressed under the control of the constitutive cauliflower mosaic virus 35S promoter in the *ah* mutant, FMTT271. A total of thirteen independent transgenic plants were obtained and verified for transgene integration by PCR using primers designed to detect the binary vector pBI121. The transgenic plant showed complementation of the *ah* phenotype, determined by visual examination of the hypocotyl color (Fig 2A and 2D). Plants from nine independent T1 generation transgenic lines, as well as the control FMTT271, were selected for further molecular and biochemical characterization, focusing particularly on leaves, although anthocyanin contents were altered throughout the transgenic plants (Fig 2A–2F). *AH* transcripts were detected in the transgenic plants and expression levels positively correlated with anthocyanin content, while neither *AH* expression nor anthocyanins were detected in FMTT271 (Fig 2G and 2H). Furthermore, transcript levels of flavonoid 3’5’-hydroxylase (*F3’5’H*, *Solyc11g066580*), dihydroflavonol 4-reductase (*DFR*, *Solyc02g085020*), anthocyanidin synthase (*ANS*, *Solyc08g080040*), flavonoid 3-O-glucosyltransferase (*3GT*, *Solyc10g083440*) and glutathione S-transferase (*GST*, *Solyc02g081340*), were all higher in the transgenic plants than in the controls (S5 Fig). These data suggested that the absence of anthocyanin in FMTT271 resulted from the loss of function of the *AH* gene. Moreover, we inferred that *AH* may control anthocyanin accumulation by up-regulating the transcript levels of anthocyanin biosynthetic genes.

### Developmental and low-temperature-induced regulation of *AH*

**Fig 3:**
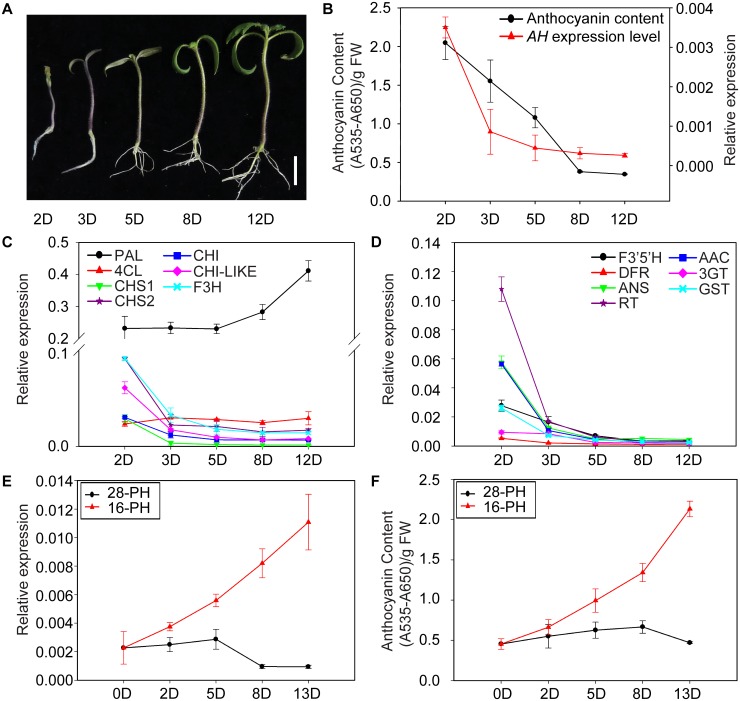
Developmental and low temperature-induced anthocyanin accumulation and expression of *AH*. (A) The phenotype of NIL-PH seedlings at five stages (2-day old to 12-day old). The seedlings were growing under normal condition (28°C/20°C, day/night). Anthocyanin content and relative expression levels of *AH* (B) and anthocyanin biosynthetic genes (C and D) in hypocotyls at different developmental stages. Hypocotyls from 20 seedlings from the five stages, respectively, were used. Relative expression levels of *AH* (E) and anthocyanin content (F) in leaves grown under 16 h of light at 16°C/ 8 h of dark at 8°C or 16 h of light at 28°C/ 8 h of dark at 20°C conditions. 5-leaf old tomato seedlings were used. The X axis indicates the duration of the different temperature treatments. Data presented are the mean of three biological replicates with error bars indicating ±SD. Scale bars, 1 cm.

Anthocyanins accumulate at different developmental stages and in different tissues [17, 21, 56]. Two-day old seedlings had a visible dark purple color with a high anthocyanin content in the hypocotyl, which was gradually lost over the subsequent 10 days (Fig 3A and 3B). Over a time course of hypocotyl development, *AH* expression was highest in 2-day old seedlings and then decreased during seedling development (Fig 3B), with a particularly marked reduction between the 2-day old and 3-day old stages. Expression of anthocyanin biosynthetic genes (except *PAL*) showed similar patterns to that of *AH* gene, with the highest levels of transcript abundance at the early stage of seedling development and a subsequent decrease to low levels (Fig 3C and 3D). These observations suggested that *AH* expression is developmentally regulated and that its expression in turn may regulate the expression of anthocyanin biosynthetic genes.

Since anthocyanin biosynthesis in tomato can be induced by exposure to low temperatures, we investigated whether *AH* expression changed in response to this stress. Five-leaf old NIL-PH plants were grown at 16°C day/8°C night conditions for 2, 5, 8, or 13 days (referred to here as 16-PH), while the control plants were grown under 28°C day/20°C night conditions (named 28-PH). *AH* transcript levels in young leaves were relatively low at the 0 day time point and then rapidly increased after exposure to low temperature conditions for 2 days, with a subsequent gradual increase over time in 16-PH plants (Fig 3E), which correlated with changes in anthocyanin content (Fig 3F). No significant changes were seen in the control plants (Fig 3E and 3F). These results suggested that *AH* functions as a regulator of anthocyanin biosynthesis in response to low temperature stress.

### Global gene regulation by *AH* in hypocotyls

To investigate the overall regulatory function of *AH* in hypocotyls, we collected the hypocotyls of 5-day old seedlings from the NIL-PH lines (named PH) and the NIL-GH lines (named GH). Two biological replicates of all samples were performed for pair-end 100 bp sequencing. In total, about 109 million clean reads were generated and 85–90% of them could be uniquely mapped to the ITAG2.4_cdna reference genome (S3 Table, Tomato Genome 2012). The unique reads were then used for the following analysis.

**Fig 4:**
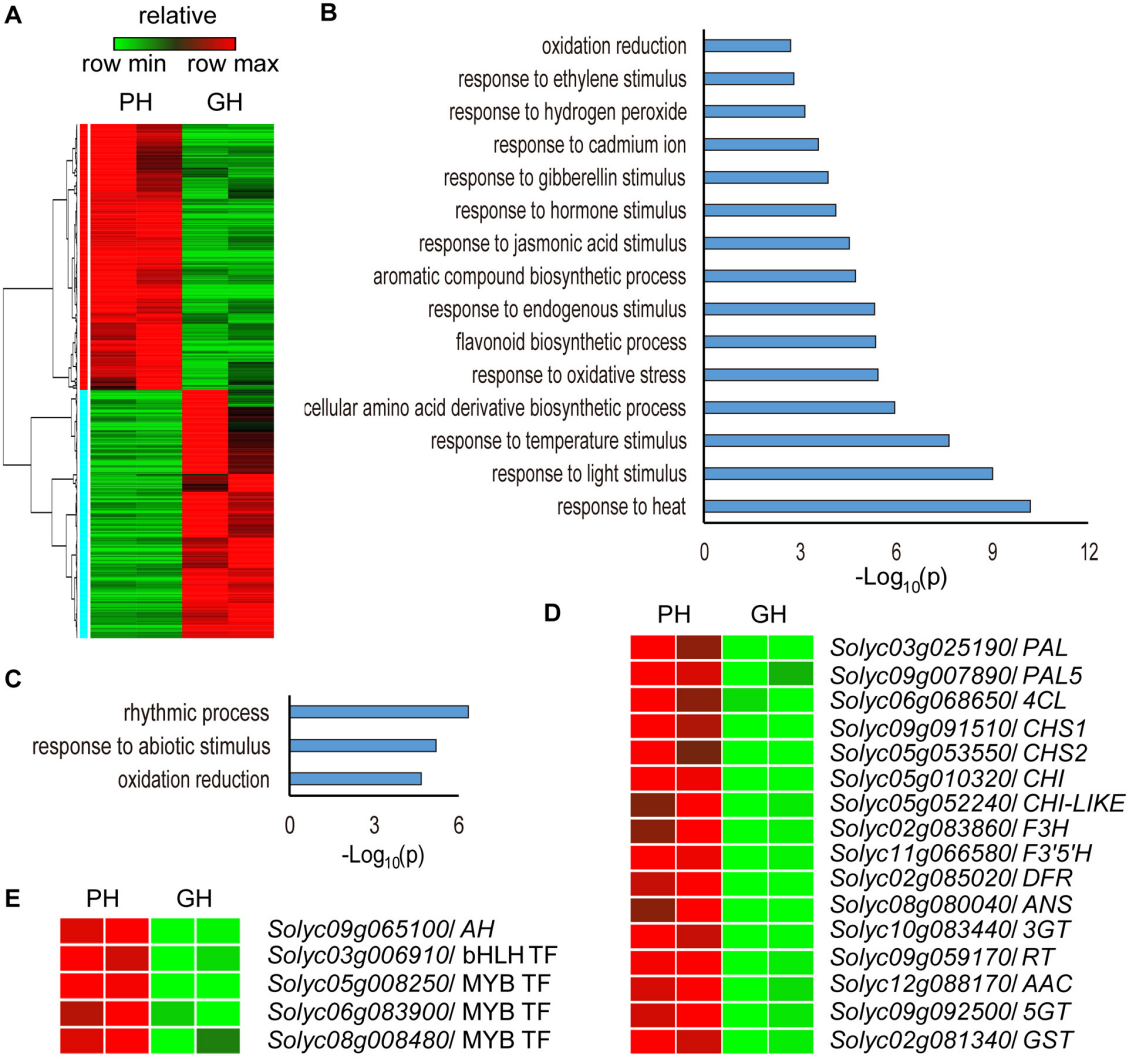
Identification of differentially expressed genes between NIL PH and GH hypocotyls. Hypocotyls from 20 5-day old seedlings from NIL PH and GH seedlings were used. Two biological replicates of all samples were performed. (A) The relative expression pattern of PH and GH differentially expressed genes. (B) The enriched GO terms in biological processes of the up-regulated genes in PH compared with GH. (C) The enriched GO terms in biological processes of the down-regulated genes in PH compared with GH. (D) The relative expression pattern of anthocyanin biosynthetic genes in PH and GH. (E) The relative expression pattern of bHLH and MYB transcription factors (TFs) among the differentially expressed genes.

Only genes with fold change (log_2_ ratio) >1 or <-1 and p value <0.01 in the comparisons were identified as DEGs. Using this cutoff, we identified 551 DEGs (S4 Table), comprising 285 that were up-regulated and 266 that were down-regulated in PH relative to GH (Fig 4A). The GO terms assigned to genes up-regulated in PH relative to GH were significantly enriched in ‘response to abiotic (heat, light stimulus, temperature stimulus, oxidative stress) stimulus’, ‘response to hormone (jasmonic acid, gibberellin, and ethylene) stimulus’ and ‘flavonoid biosynthetic process’ (Fig 4B), while the down-regulated genes were enriched in ‘response to abiotic stimulus’, ‘oxidation reduction’ and ‘rhythmic process’ (Fig 4C).

Consistent with the anthocyanin content in PH and GH, the transcripts of anthocyanin biosynthetic genes, including *PAL* (*Solyc03g025190*), *PAL5* (*Solyc09g007890*), *4CL* (*Solyc06g068650*), *CHS1* (*Solyc09g091510*), *CHS2* (*Solyc05g053550*), *CHI* (*Solyc05g010320*), *CHI-LIKE* (*Solyc05g052240*), *F3H* (*Solyc02g083860*), *F3'5'H* (*Solyc11g066580*), *DFR* (*Solyc02g085020*), *ANS* (*Solyc08g080040*), *3GT* (*Solyc10g083440*), *RT* (*Solyc09g059170*), *AAC* (*Solyc12g088170*), *5GT* (*Solyc09g092500*) and *GST* (*Solyc02g081340*), were up-regulated in PH compared with GH (Fig 4D). The *AH* transcript levels were also much higher in PH than in GH (Fig 4E), and one bHLH TF (*Solyc03g006910*) and three R2R3-MYB TFs (*Solyc05g008250*, *Solyc06g083900* and *Solyc08g008480*) were both found amongst the DEGs, with much higher transcript levels in PH than in GH (Fig 4E). To validate the RNA-seq expression data, we assessed the transcript levels of 7 known anthocyanin biosynthetic genes as well as a randomly-selected gene (*Solyc06g049020*) by qPCR and found that the qPCR data were consistent with those obtained from the RNA-seq experiment (S6 Fig).

### Global gene regulation by *AH* in seedlings under low temperature stress

To investigate the function of *AH* in tomato seedlings under low temperature stress, we performed two comparative transcriptome analyses: 1) 5-leaf old seedlings of NIL-PH and NIL-GH lines were grown under normal conditions (28°C/20°C, day/night) and then exposed to a subsequent stress of 16°C/8°C (day/night) for 5 days, named 16-PL and 16-GL, respectively; and 2) 5-leaf old seedlings of NIL-PH and NIL-GH lines were grown under normal conditions (28°C/20°C, day/night) through the duration of the experiment, named 28-PL and 28-GL, respectively. RNA was extracted from leaves of individual lines and used for RNA-seq profiling. Two biological replicates of all samples were analyzed. In total, approximately 207.1 million clean reads were generated, 78–90% of which could be uniquely mapped to the ITAG2.4_cdna reference genome (S3 Table, Tomato Genome 2012). The unique reads were then used for the following analysis.

**Fig 5:**
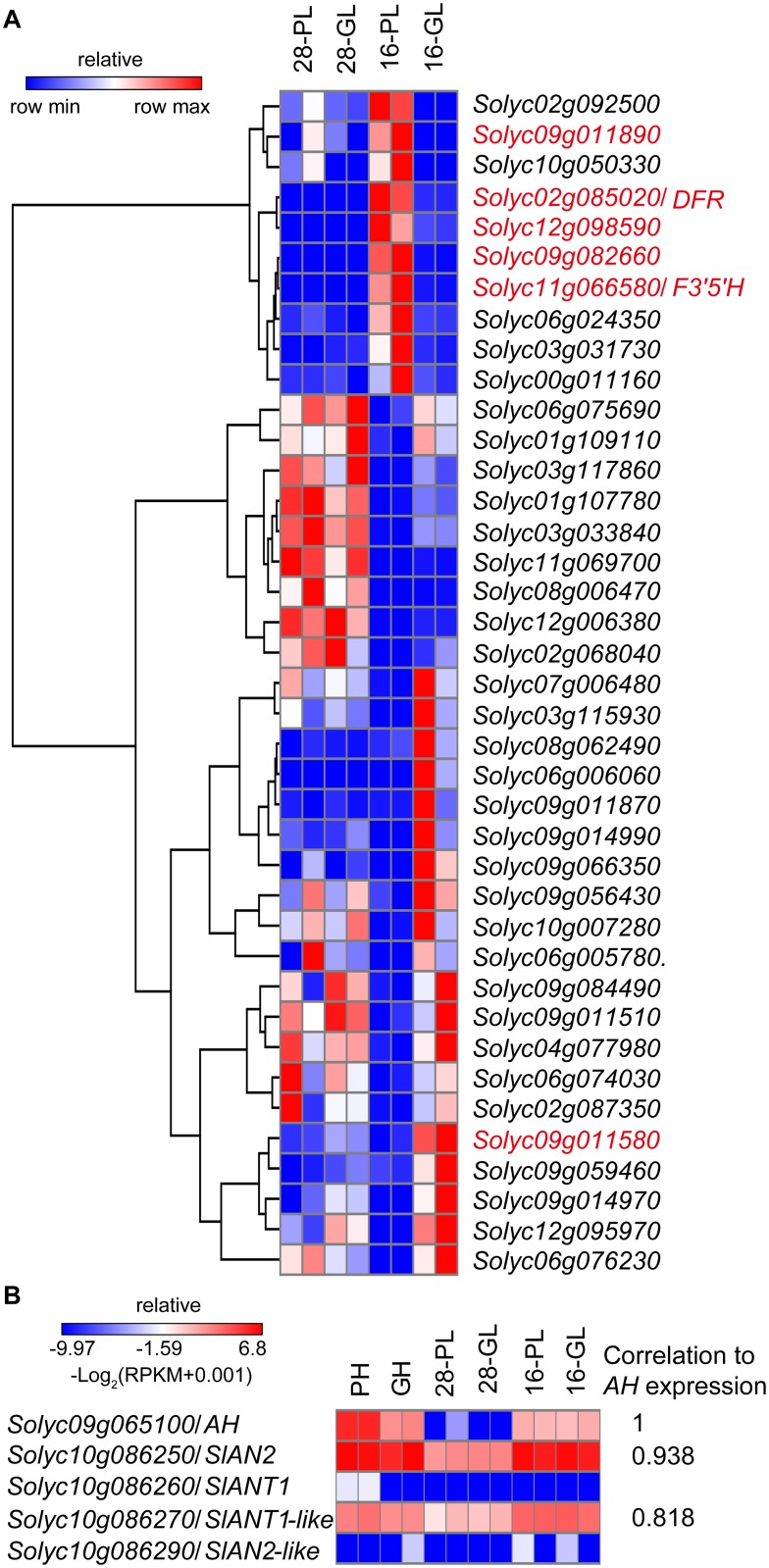
Identification of differentially expressed genes between NIL 16-PL and 16-GL seedlings under low temperature stress. Five-leaf old NIL PH and GH seedlings grown at 28°C were treated at 28°C/20°C (day/night, 16h/8h, named 28-PL and 28-GL, respectively) or 16°C/8°C (day/night, 16h/8h, named 16-PL and 16-GL, respectively) for 5 days. RNA was extracted from leaves of individual lines and used for RNA-seq profiling. Two biological replicates of all samples were prepared. (A) The relative expression pattern of differentially expressed genes between 16-PL and 16-GL seedlings under low temperature conditions, excluding the differentially expressed genes from the 28-PL and 28-GL comparison. The genes in red color mean that the same expression pattern of these genes were observed in NIL-PH relative to NIL-GH. (B) The relative expression pattern of anthocyanin regulator genes *AH*, *SlAN2*, *SlANT1*, *SlANT1-like* and *SlAN2-like* in all samples. The correlation value of *SlAN2*, *SlANT1-like* to *AH* was evaluated by the normalized RPKM (log_2_(RPKM+0.001)) from the 6 samples, respectively.

Using the same cutoff as was that which used for the hypocotyls, 75 and 77 DEGs were identified in the 16-PL versus 16-GL and 28-PL versus 28-GL comparisons, respectively. However, in both the RNA-seq and qPCR data, no transcripts of *AH* were detected in the 28-PL and 28-GL samples (S6 Fig), suggesting that the DEGs between 28-PL and 28-GL were caused by other genes from the segment introgressed along with *AH*. After filtering out the DEGs from the 28-PL versus 28-GL comparison, 39 DEGs were left in 16-PH compared with 16-GH (S5 Table). Among the 39 DEGs, 10 genes were up-regulated in 16-PL relative to 16-GL, including five genes (*Solyc09g011890*, *Solyc02g085020* (*DFR*), *Solyc12g098590*, *Solyc09g082660*, *Solyc11g066580* (*F3'5'H*)) that were also up-regulated in PH relative to GH (Fig 5A). The others showed a down-regulation, including one gene *(Solyc09g011580*) that was also down-regulated in PH relative to GH (Fig 5A). GO enrichment analysis showed that the up-regulated genes were significantly enriched in the terms ‘phenylpropanoid biosynthetic process’, ‘cellular amino acid derivative biosynthetic process’ and ‘aromatic compound biosynthetic process’, while the down-regulated genes were enriched in the ‘response to carbohydrate stimulus’ category (S6 Table).

Previous studies identified four tomato MYB TFs (*Solyc10g086250* (*SlAN2*), *Solyc10g086260* (*SlANT1*), *Solyc10g086270* (*SlANT1-like*), *Solyc10g086290* (*SlAN2-like*)) that regulate anthocyanin biosynthesis [36, 42, 43]. To investigate which MYB regulator may be the best candidate protein to work in combination with AH, the transcripts of these five genes were analyzed in the 12 samples. No *SlANT1* and *SlAN2*-*like* transcripts were detected in any of the samples (Fig 5B). The correlation of transcript levels in all samples of *SlAN2* and *SlANT1*-*like* to *AH* were 0.938 and 0.818, respectively, suggesting that they exhibit a similar expression pattern to *AH* (Fig 5B).

### The *ah* mutation causes the accumulation of reactive oxidative species and constitutively activate defense responses under cold conditions

**Fig 6:**
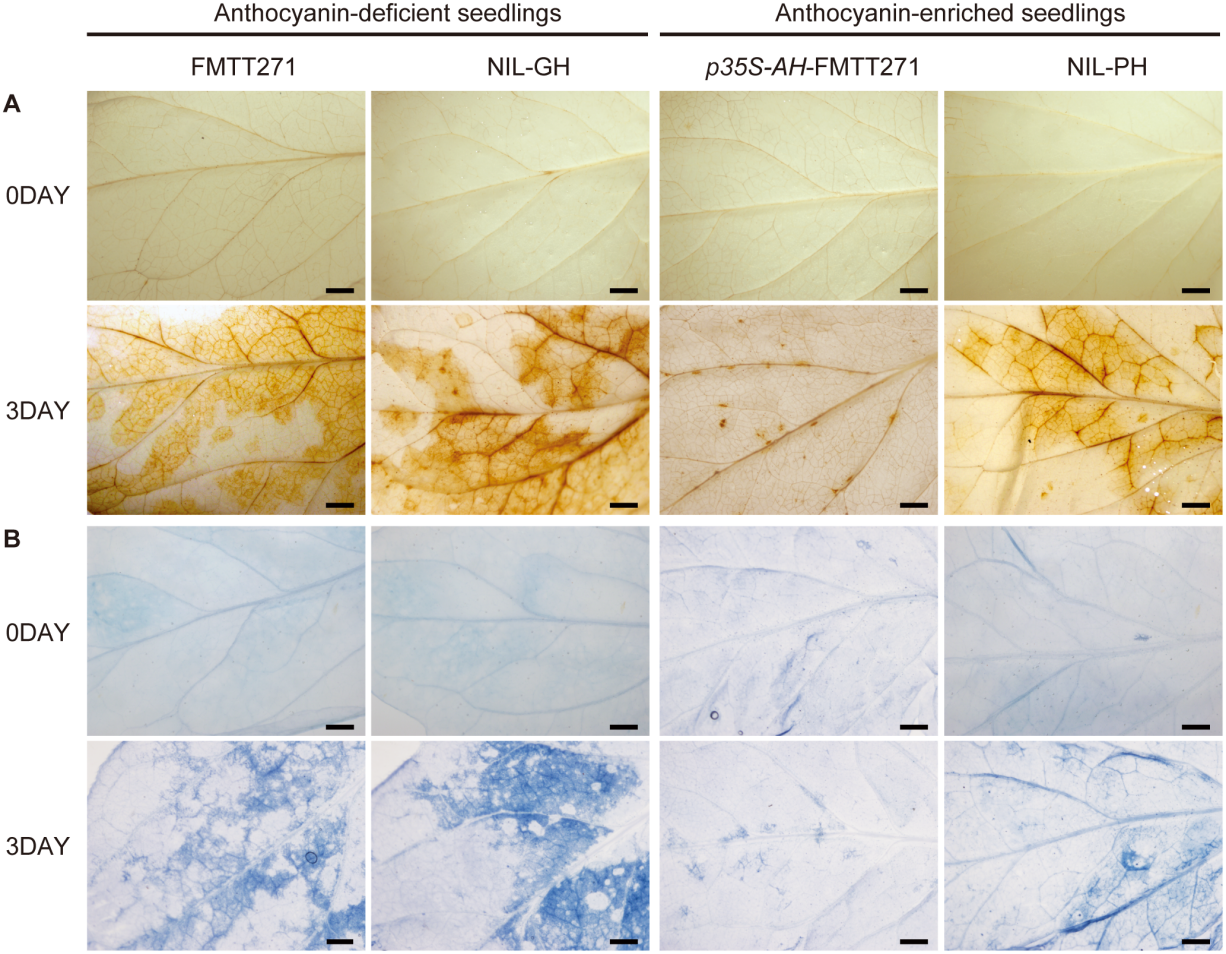
Phenotypes of anthocyanin deficient and anthocyanin enriched plants under cold stress. Five-leaf old plants were treated at 4°C for 0 days and 3 days. (A) H_2_O_2_ accumulation in the leaves of the anthocyanin deficient plants, FMTT271 and NIL-GH, and the anthocyanin-enriched plants, NIL-PL and *p35S-AH*-FMTT271, as determined by DAB staining. (B) Cold-induced cell death in the mutant, FMTT271, NIL-GH and wild-type NIL-PL and p35S-*AH*-FMTT271 plants. Detached leaves were stained with trypan blue. Images are of representative plants. Scale bars, 0.2 cm.

Low temperature can perturb electron transport chains and cause the production of reactive oxidative species (ROS) [57]. To investigate whether the changes in *AH* expression can cause differences in ROS accumulation between the anthocyanin-deficient seedlings (including FMTT271 and NIL-GH) and anthocyanin-enriched seedlings (including *p35S-AH*-FMTT271 and NIL-PH) under cold stress, the presence of hydrogen peroxide (H_2_O_2_) in the leaves of the four lines (FMTT271, NIL-GH, *p35S-AH*-FMTT271 and NIL-PH) was assessed using DAB staining. Strong staining was detected in cold-treated anthocyanin-deficient plants (FMTT271 and NIL-GH) but not in the transgenic plants (*p35S-AH*-FMTT271; Fig 6A). In addition, staining in NIL-PH plants was also detected, but at very low concentrations compared with the anthocyanin-deficient seedlings (Fig 6A), consistent with low anthocyanin content and transcript levels of *AH* in NIL-PH leaves (Fig 1C and 1H). These results indicated that *ah* mutant accumulated more H_2_O_2_ than the wild-type plants under cold stress.

ROS accumulation may cause significant damage to cellular components, such as membrane lipids [57]. Trypan Blue staining is performed to investigate the differences of membrane damage or deterioration between the anthocyanin-deficient seedlings and anthocyanin-enriched seedlings under cold stress. Extensive cell death occurred in the cold-treated anthocyanin-deficient plants, but not in the anthocyanin-enriched plants (*p35S-AH*-FMTT271 and NIL-PH), as revealed by trypan blue staining (Fig 6B), indicating that membrane damage was less serious in anthocyanin-enriched plants than that in anthocyanin-deficient plants.

## Discussion

### *AH* encodes a bHLH TF that is important for anthocyanin biosynthesis

Here, we characterized an *ah* mutant, FMTT271, which lacks visible purple color in all organs throughout its life cycle, from the seedling to the flowering stages (S1 Fig), and confirmed that the green phenotype of *ah* was caused by an anthocyanin deficiency (Fig 1B). Notably, the purple phenotype of the wild-type plants is associated primarily with young tissues (S1 Fig), as has been observed in other plant species [32, 56, 58]. We propose that high anthocyanin content in younger organs that may be more vulnerable to abiotic stress may play an important role in providing protection from changing environments.

*AH* encodes a bHLH protein with high sequence homology to petunia AN1 and TT8 (Fig 1I), and is a member of the sub-group IIIf of the bHLH family. Amino acid sequence alignments showed that it contains three conserved regions, including a MYB interaction domain at the N-terminus, a bHLH domain and an ACT-like domain at the C-terminus (S3 Fig). Studies of *A*. *thaliana* have demonstrated that the N-terminal region of group IIIf proteins, which contains the acidic domain, is sufficient for trichome and non-hair root cell differentiation, but not for the induction of anthocyanin biosynthesis [59]. The ACT domain is a small molecule binding region that interacts with ligands and functions in the regulation of metabolism, solute transport and signal transduction [60]. The ACT-like domain of the maize Lc protein was shown to mediate homo-dimer formation and to be critical for the regulatory activity of Lc [61]. In this study, we found that the *ah* mutant harbors a single-base substitution (T→G at position 550 bp from the ATG start codon) in the *AH* coding sequence (Fig 1G), which is predicted to cause the introduction of a stop codon in place of a Gly, thereby truncating the predicted protein by 501 amino acids (S3 Fig). This deleted region includes both the bHLH and ACT-like domains, which likely explains the anthocyanin deficiency in the *ah* mutant. In addition, the difference in *AH* expression between NIL PH and GH suggests that the SNPs or inserts/deletions in the putative promoter may affect regulation of the gene (S4 Fig). Alternatively, it is possible that a functional *AH* positively regulates its own expression, as was reported to be the case for *TT8* [62].

Previous studies have shown that overexpression of bHLH TFs in tomato can significantly enhance the anthocyanin accumulation in leaves and fruits [19, 63, 64]. Here, overexpression of *AH* in tomato significantly enhanced anthocyanin levels in hypocotyls, leaves and fruit peels (Fig 2A–2F), and the elevated anthocyanin content in leaves was associated with an increased expression of *AH* (Fig 2G and 2H). *AH* over-expression resulted in the up-regulation of almost all LBGs (S5 Fig), which is consistent with the results of the RNA-seq analyses of the hypocotyls (Fig 4D), where *F3’5’H*, *DFR*, *ANS*, *3GT* and *GST* were all co-expressed with *AH* in purple tissues. However, in the RNA-seq analyses, under low temperature stress, only the LBGs, *DFR* and *F3’5’H* were always co-expressed with *AH*, suggesting that *AH* may directly regulate the expression of *DFR* and *F3’5’H*, which is in accordance with studies of other plant species [17, 65]. In *A*. *thaliana*, *DFR* is co-expressed with *TT8*, suggesting that TT8 may control *DFR* expression [11]. In petunia, *AN1* was also shown to be involved in the transcriptional activation of *DFR* [17]. Furthermore, in apple (*Malus domestica*), MdbHLH3, which has high homology to TT8, binds to the promoters of *DFR* and *3GT* to activate their transcription [65]. We also noticed that all of the anthocyanin biosynthetic genes, including EBGs and LBGs, were up-regulated in the hypocotyls of NIL-PH compared to NIL-GH (Fig 4D), while only LBGs, including *DFR* and *F3’5’H*, were up-regulated in the leaves of NIL-PH compared to NIL-GH (S5 Fig and Fig 5A). These results suggest that the regulatory mechanism controlling anthocyanin biosynthesis may differ between organs, as has been observed in *A*. *thaliana* [28].

### *AH* controls developmental and low-temperature-induced anthocyanin pigmentation patterns

Previous studies have suggested that both developmentally controlled and tissue-specific patterns of anthocyanin accumulation are primarily regulated by the activity of R2R3-MYB TFs in the MYB-bHLH-WD40 (MBW) complex [66]. Recently, the expression of several bHLH TFs has been shown to be up-regulated during development, accompanying anthocyanin-related color changes. For example, the expression of tobacco *NtAn1a* was shown to be up-regulated during flower development [67], as was the expression of petunia *AN1* [23, 68]. This suggests that bHLH TFs may act together with R2R3-MYB TFs or WD-40 proteins in the developmental regulation of anthocyanin biosynthesis. Most to date have focused on reproductive organs, including flowers and fruits, while in this study we focused on hypocotyls of young seedlings. We found that *AH* transcript levels in hypocotyls decreased from day 2 to day 12 after germination (Fig 3B), in parallel with a similar expression pattern of anthocyanin biosynthetic genes (Fig 3C and 3D). Meanwhile, the purple color of the hypocotyl became less prominent during seedling development (Fig 3A and 3B). Consistent with a regulatory role in anthocyanin biosynthesis, we hypothesized that the reduction in *AH* expression might underlie the corresponding decrease of transcripts of the anthocyanin biosynthetic genes and anthocyanin abundance.

In addition to developmental regulation, there is growing evidence that members of the MBW complex, are involved in environmental responses. For example, high-light-induced anthocyanin production in petunia is associated with increased expression of the bHLH factor, *AN1*, and two R2R3-MYB factors, *PHZ* and *DPL* [68]. In *A*. *thaliana*, *TT8* expression is induced by high light conditions and suppressed by high temperatures [69], and expression of the apple bHLH gene, *MdbHLH3*, is induced by low temperatures at both the transcriptional and translational levels [65].

Tomato is a warm season crop that grows best at daytime temperatures of 25–28°C, and most commercial tomato cultivars are sensitive to low temperatures during all stages of development [70]. Here, we assessed the anthocyanin content of leaves when the temperature was lowered from 28°C to 16°C. Our results were consistent with a report showing that low temperatures induce expression of anthocyanin biosynthetic genes in tomato leaves, resulting in increased anthocyanin accumulation [71], and we investigated the underlying molecular mechanisms. *AH* transcript levels in NIL PH plants were low prior to treatment, but increased substantially after growth in low temperature conditions for 2 days, followed by a gradual increase over time (Fig 3E). These results are consistent with the transcriptome analyses (Fig 4B), suggesting that *AH* responded to the low temperature stress by inducing the expression of anthocyanin biosynthetic genes to increase anthocyanin accumulation. This provides additional evidence supporting the involvement of bHLH proteins in the MBW complex during responses to environmental stresses [69]. A recent study reported that among SlANT1, SlAN2, SlANT1-like and SlAN2-like, only SlAN2 acts as a positive regulator of anthocyanin synthesis in vegetative tissues under high light or low temperature conditions [36]. Further, it is suggested that the SlAN2, together with SlAN1 and probably SlJAF13, is involved in the regulation of anthocyanin biosynthesis is tomato vegetable tissues under cold induction [36]. Indeed, we detected the same expression pattern of *AN2* and *ANT1-like* and *AH*, both in hypocotyls and seedlings (Fig 5B), suggesting that AH might combine with AN2 or ANT1-like, but not ANT1, to regulate anthocyanin biosynthesis in vegetative tissues.

### *AH* expression in tomato seedlings promotes low temperature tolerance

Using DAB and trypan blue staining assays, we found that anthocyanin-deficient seedlings have increased ROS accumulation and constitutively activated defense responses compared to anthocyanin-enriched seedlings under low temperature stress (Fig 6), suggesting that the anthocyanin-enriched plants have a higher tolerance of low temperatures. In addition, from the RNA-seq analyses, we observed increased transcript levels of not only genes involved in anthocyanin biosynthesis in anthocyanin-enriched plants, but also those of genes related to several other processes, based on the GO term analyses. This suggests that AH has a greater range of functions than would be deduced based on genetic analyses of homologs from other plants species. Interestingly, the GO term analyses showed that the up-regulated genes in PH relative to GH were significantly enriched not only in the flavonoid biosynthesis, but also in ‘response to abiotic stimulus’ (Fig 4B). Flavonoids, including anthocyanin, are well known to provide protection against biotic and abiotic stresses [2–6]. That the GO terms were enriched in ‘response to abiotic stimulus’ suggests that the young, more vulnerable seedlings adjusted in order to meet the changed environments, to minimize damage from biotic or abiotic stresses, such as chilling. These observations have also been made in studies of *Betula pendula* and *Vaccinium spp*. [72, 73], and in tomato, the genes involved in ‘stress responses’ were also found to be up-regulated in the purple fruit peel of *Aft*/*Aft atv*/*atv* plants [74]. Recently, several studies revealed that anthocyanin-enriched tomato fruits have substantially increased shelf life, as evidenced by delayed over-ripening and reduced susceptibility to the fungal pathogen, *Botrytis cinerea* [7, 75]. In addition, overexpression of *SlAN2* in tomato resulted in a decrease in the levels of ROS under heat stress and enhanced thermo-tolerance [76]. Thus, we propose that the high resistance of anthocyanin-enriched plants to environmental stresses is not only due to the TFs regulating the anthocyanin content, but also to the regulation of genes associated with environmental stresses.

### Conclusion

There is considerable interest in understanding the mechanisms underlying the regulation of anthocyanin biosynthesis in vegetable crop species, due to the importance of anthocyanins in plant stress tolerance and their value in the human diet. Here, we identified a bHLH TF gene, *AH*, by map-based cloning. We suggest that *AH* controls anthocyanin accumulation by up-regulating transcript levels of anthocyanin biosynthetic genes. In addition, we found that *AH* transcription increased during developmental and was induced by low temperature stimuli. Transcriptome analyses of the hypocotyls and leaves revealed that AH may not only regulate the expression of genes involved in anthocyanin biosynthesis, but also genes involved in responses to abiotic stimuli. Moreover, compared to *AH* overexpression lines, the *ah* mutant showed increased accumulation of ROS and constitutively activated defense responses under cold conditions, suggesting that *AH* plays a key role in providing protection against low-temperature stress.

## Supporting Information

S1 Fig Visual phenotypic comparison of the wild-type NIL-PH (purple hypocotyl lines) and mutant NIL-GH (green hypocotyl lines) from the NIL population. (A) Young seedlings of 5-day-old plants. (B) Young leaves. (C) Buds. (D) Young sepals. (E) Petals. Scale bars, 1 cm (A, C), 0.5 cm (B, D), 0.1 cm (E).
(TIF)

S2 Fig The phenotypes of the *ah* mutant LA0260 and F_1_ hybrids from crosses between FMTT271 and LA0260. (A) Young seedlings of LA0260, F_1_ hybrids from cross between FMTT271 and LA0260. (B) Nucleotide alignment showing part of the six exons of *AH* in LA716, Heinz1706, FMTT271 and LA0260.
(TIF)

S3 Fig Multiple alignment analysis of selected bHLH proteins. Alignment of deduced amino acid sequences of AH with bHLH homologs from *Nicotiana tabacum* (tobacco AN1b), *Petunia×hybrida* (Petunia AN1), *Zea mays* (Maize Lc), and *Arabidopsis thaliana* (*Arabidopsis* TT8). The N-terminal Myb interaction region (MIR), bHLH domain and the putative ACT-like domain at the C-termini are indicated by straight lines. The arrow indicates the *ah* mutant substitution site.
(TIF)

S4 Fig Alignment analysis of the putative promoter of *AH* between LA716 and FMTT271. The sequences of -984 bp relative to the start codon of *AH* were used as putative promoter.
(TIF)

S5 Fig qPCR analysis of anthocyanin biosynthetic genes in leaves of control and transgenic lines. Relative transcript levels of early anthocyanin pathway biosynthetic genes (A) and late anthocyanin pathway biosynthetic genes (B).
(TIF)

S6 Fig Confirmation of RNA-seq results by qPCR. Seven anthocyanin-related genes (*AH*, *CHS1*, *F3’5’H*, *ANS*, *RT*, and *AAC*) and a randomly-selected gene (*Solyc06g049020*) were analyzed. The tomato *ACTIN* (*Solyc03g078400*) gene was used as the reference gene, and all of the analyses were performed with three technical replicates.
(TIF)

S1 Table Genetic segregation analysis of the green locus of FMTT271 in the backcross (BC) population. (PDF)

S2 Table *AH* candidate genes. (PDF)

S3 Table Raw RNA-seq data and mapping statistics. (PDF)

S4 Table The 551 DEGs between PH and GH. (XLS)

S5 Table The 39 DEGs between 16-PH and 16-GH. (XLS)

S6 Table The enriched GO terms in the biological processes category amongst the DEGs identified in 16-PH compared with 16-GH. (PDF)

S7 Table Primers used in this study. (PDF)
