# Supplementary figures and images for: The Tomato Hoffman’s Anthocyaninless Gene Encodes a bHLH Transcription Factor Involved in Anthocyanin Biosynthesis That Is Developmentally Regulated and Induced by Low Temperatures
Source: PLoS One. 2016 Mar 4;11(3):e0151067. doi: 10.1371/journal.pone.0151067 (PMC4778906; doi:10.1371/journal.pone.0151067)

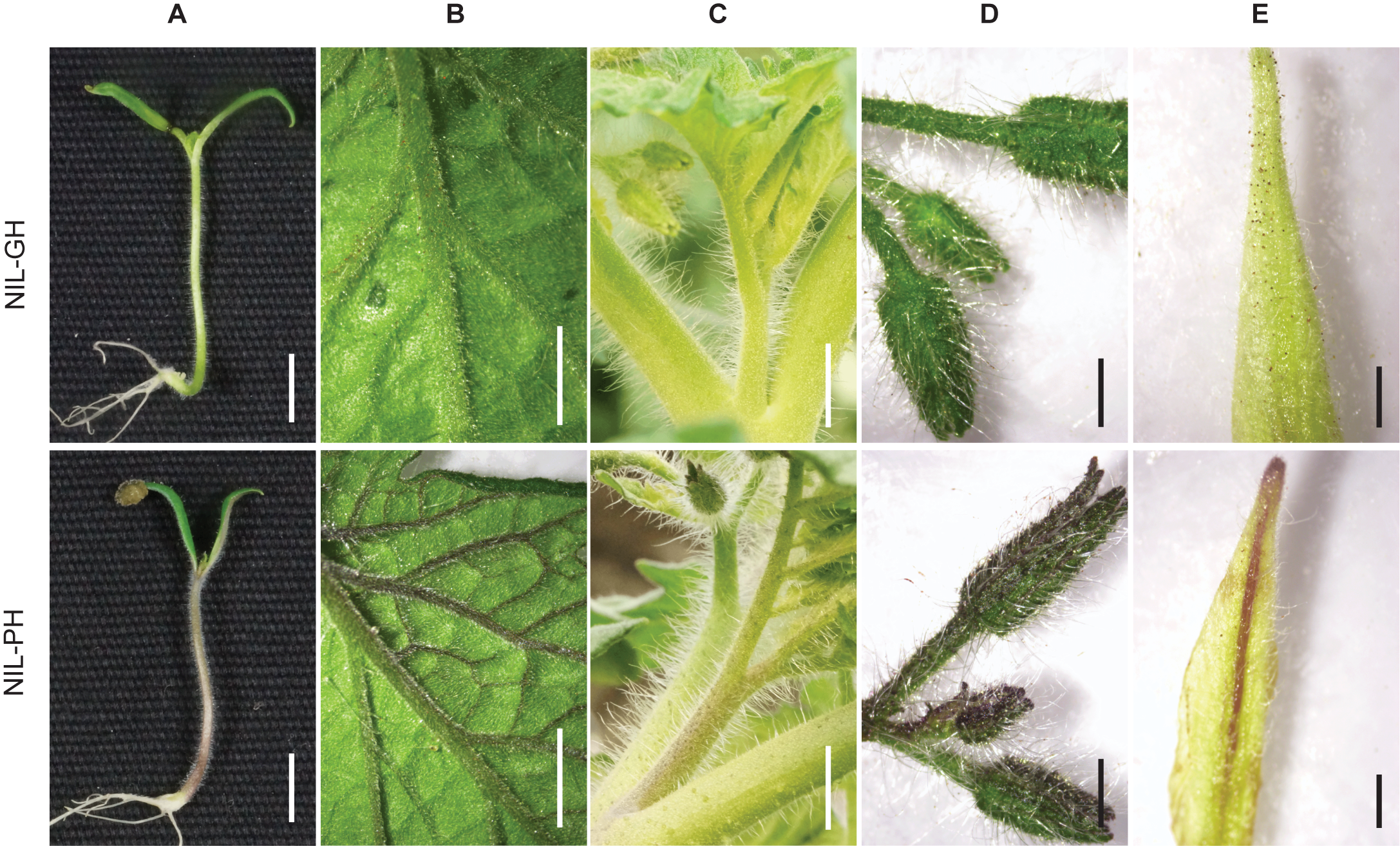

Supplement: S1 Fig — (A) Young seedlings of 5-day-old plants. (B) Young leaves. (C) Buds. (D) Young sepals. (E) Petals. Scale bars, 1 cm (A, C), 0.5 cm (B, D), 0.1 cm (E). (TIF) [file pone.0151067.s001.tif]

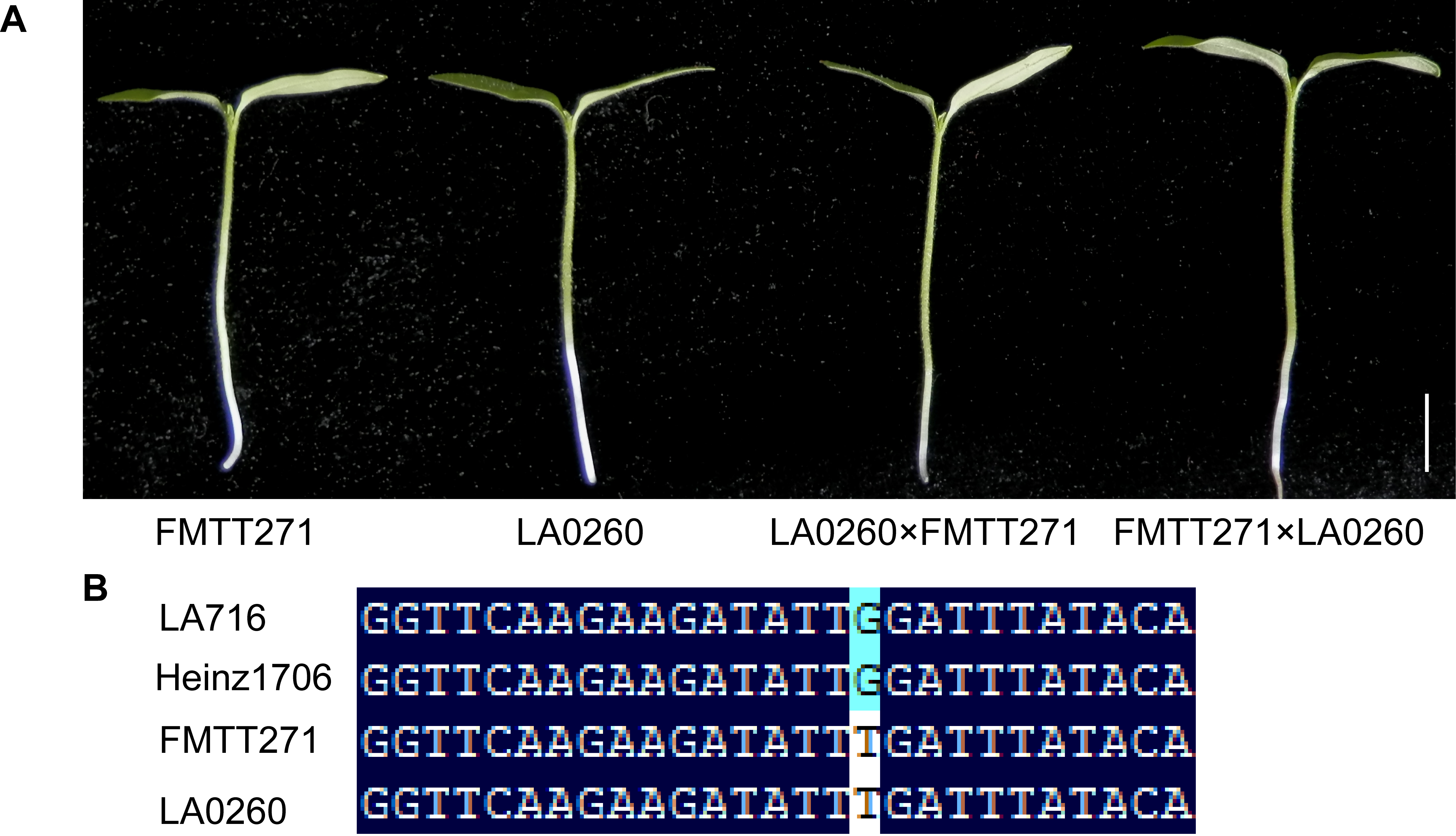

Supplement: S2 Fig — (A) Young seedlings of LA0260, F1 hybrids from cross between FMTT271 and LA0260. (B) Nucleotide alignment showing part of the six exons of AH in LA716, Heinz1706, FMTT271 and LA0260. (TIF) [file pone.0151067.s002.tif]

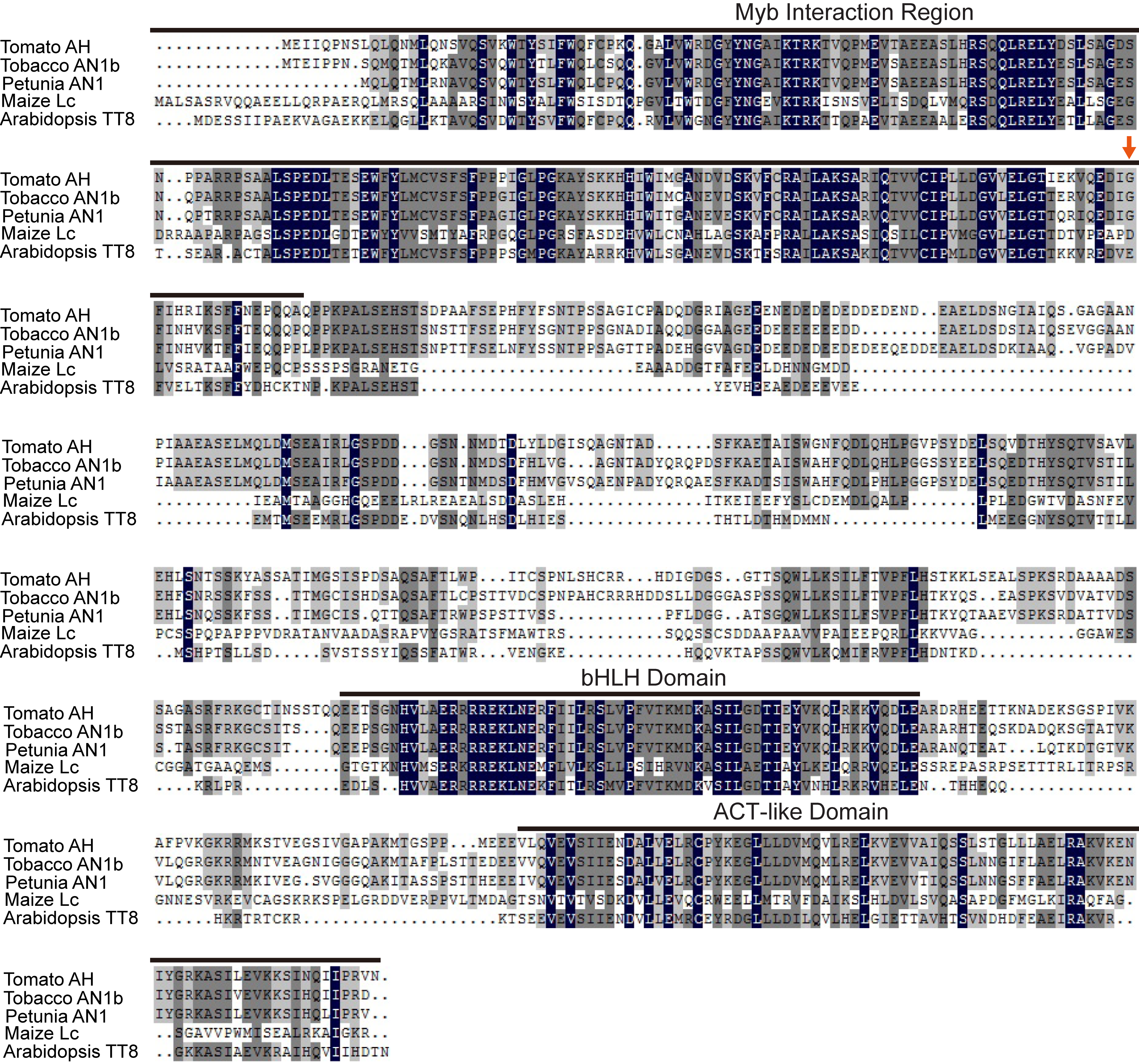

Supplement: S3 Fig — Alignment of deduced amino acid sequences of AH with bHLH homologs from Nicotiana tabacum (tobacco AN1b), Petunia×hybrida (Petunia AN1), Zea mays (Maize Lc), and Arabidopsis thaliana (Arabidopsis TT8). The N-terminal Myb interaction region (MIR), bHLH domain and the putative ACT-like domain at the C-termini are indicated by straight lines. The arrow indicates the ah mutant substitution site. (TIF) [file pone.0151067.s003.tif]

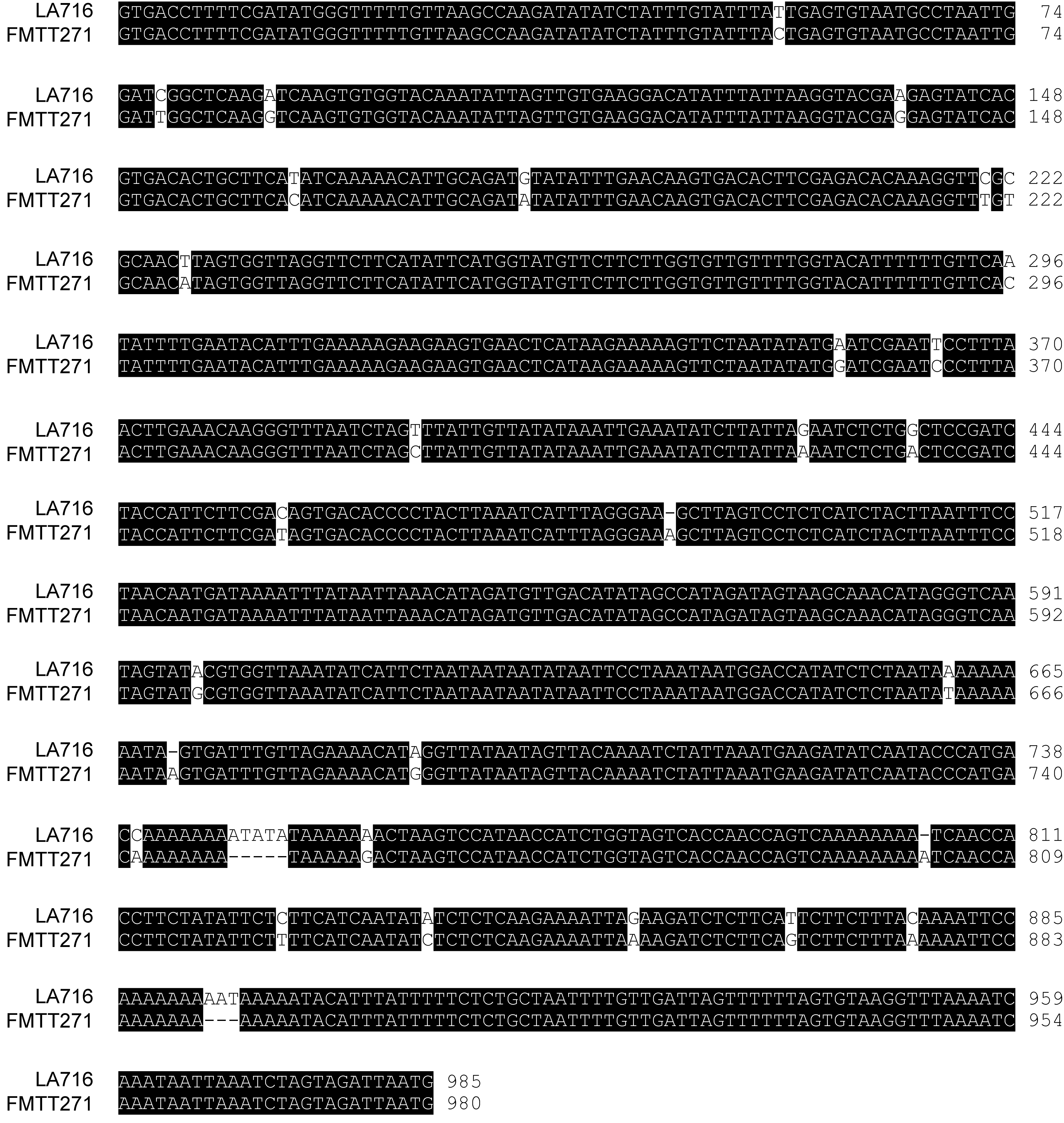

Supplement: S4 Fig — The sequences of -984 bp relative to the start codon of AH were used as putative promoter. (TIF) [file pone.0151067.s004.tif]

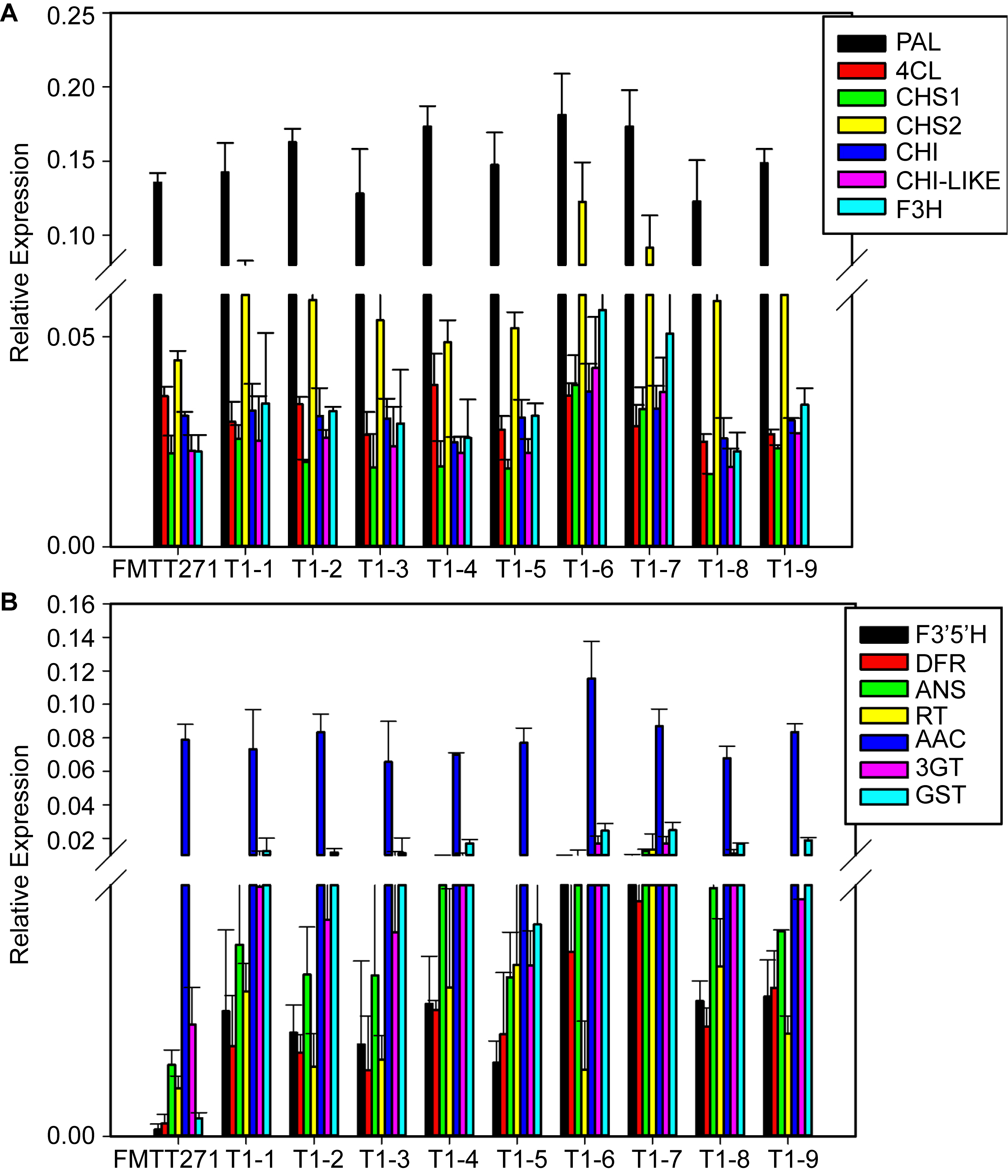

Supplement: S5 Fig — Relative transcript levels of early anthocyanin pathway biosynthetic genes (A) and late anthocyanin pathway biosynthetic genes (B). (TIF) [file pone.0151067.s005.tif]

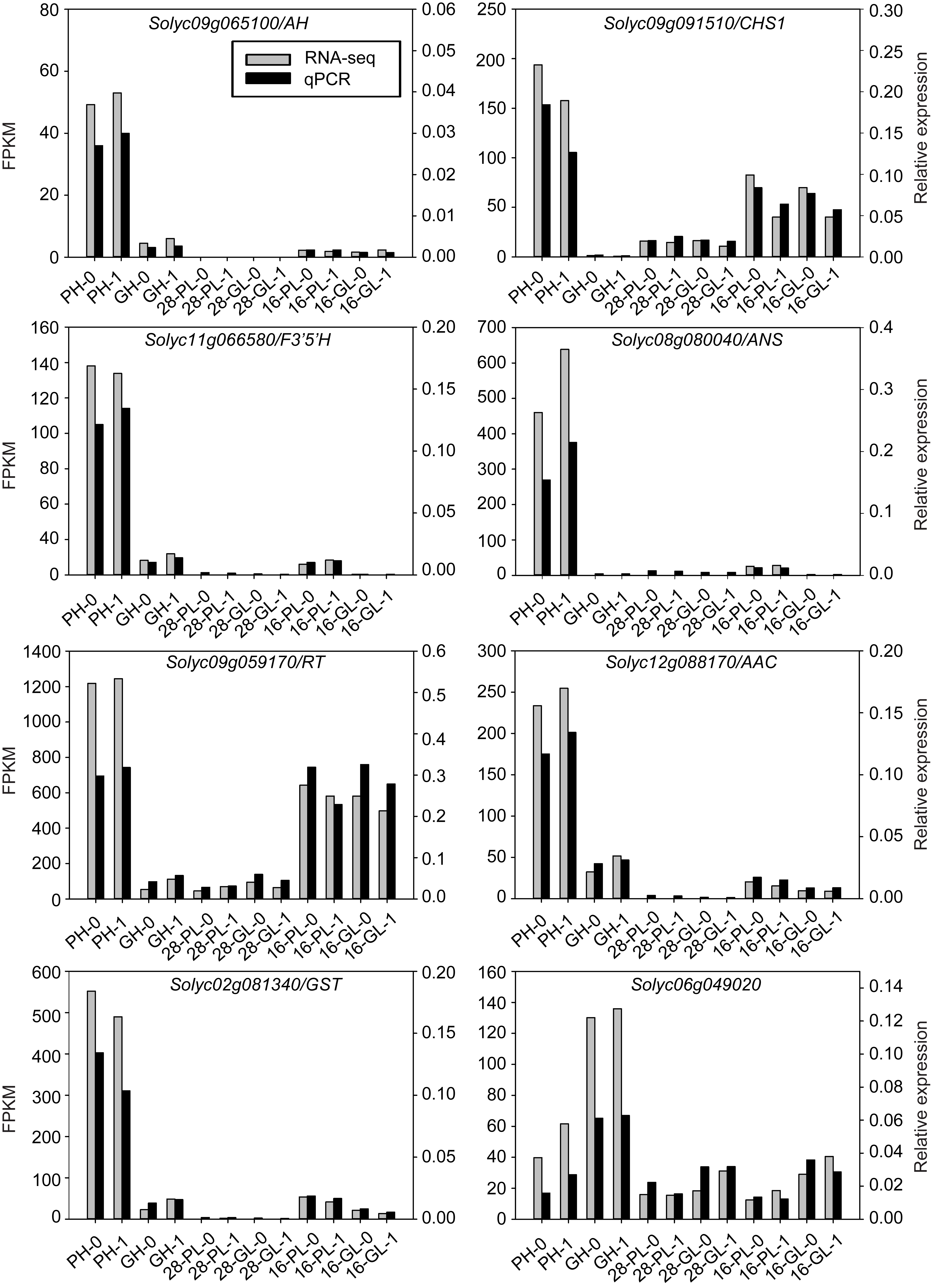

Supplement: S6 Fig — Seven anthocyanin-related genes (AH, CHS1, F3’5’H, ANS, RT, and AAC) and a randomly-selected gene (Solyc06g049020) were analyzed. The tomato ACTIN (Solyc03g078400) gene was used as the reference gene, and all of the analyses were performed with three technical replicates. (TIF) [file pone.0151067.s006.tif]
