# Supplementary material for: The Tomato Hoffman’s Anthocyaninless Gene Encodes a bHLH Transcription Factor Involved in Anthocyanin Biosynthesis That Is Developmentally Regulated and Induced by Low Temperatures
Source: PLoS One. 2016 Mar 4;11(3):e0151067. doi: 10.1371/journal.pone.0151067 (PMC4778906; doi:10.1371/journal.pone.0151067)
Supplement: S1 Table — (PDF) [file pone.0151067.s007.pdf]

**S1 Table. Genetic segregation analysis of green locus in FMTT271 in the backcross (BC) population.**

| BC population | Total No. | No. of purple plants[a] | No. of green plants | Radio | χ2 test |
|---|---|---|---|---|---|
| BC1 | 12 | 5 | 7 | 0.71:1 | 0.667* |
| $BC_4F_1$ | 719 | 549 | 170 | 3.23:1 | 0.705* |

[a]Purple plant and green plants were determined by visual inspection.

*P-value<0.05 (highly significant).
