# Supplementary material for: The Tomato Hoffman’s Anthocyaninless Gene Encodes a bHLH Transcription Factor Involved in Anthocyanin Biosynthesis That Is Developmentally Regulated and Induced by Low Temperatures
Source: PLoS One. 2016 Mar 4;11(3):e0151067. doi: 10.1371/journal.pone.0151067 (PMC4778906; doi:10.1371/journal.pone.0151067)
Supplement: S2 Table — (PDF) [file pone.0151067.s008.pdf]

S2 Table. Candidate genes of *AH*.

Tomato geneID	Arabidopsis_homology	Arabidopsis_genename	Arabidopsis_description
Solyc09g065060	AT1G49920	T18C15_1	MuDR family transposase
Solyc09g065070	AT5G46600	ALMT13	Aluminium activated malate transporter family protein
Solyc09g065080	AT1G71800	CSTF64	cleavage stimulating factor 64
Solyc09g065090	AT1G71800	CSTF64	cleavage stimulating factor 64
Solyc09g065100	AT4G09820	TT8	basic helix-loop-helix (bHLH) DNA-binding superfamily protein
