# Supplementary material for: The Tomato Hoffman’s Anthocyaninless Gene Encodes a bHLH Transcription Factor Involved in Anthocyanin Biosynthesis That Is Developmentally Regulated and Induced by Low Temperatures
Source: PLoS One. 2016 Mar 4;11(3):e0151067. doi: 10.1371/journal.pone.0151067 (PMC4778906; doi:10.1371/journal.pone.0151067)
Supplement: S3 Table — (PDF) [file pone.0151067.s009.pdf]

S3 Table. Raw data and mapping statistic.

Sample	Clean reads	Uniquely mapped reads	Uniquely mapped reads rate
PH-1	34379393	29346119	85%
PH-2	24465161	21048348	86%
GH-1	24971082	22460295	90%
GH-2	25224425	22292506	88%
28-PL-1	27210895	23112092	85%
28-PL-2	22032767	19194487	87%
28-GL-1	27244034	21438769	79%
28-GL-2	26760934	23998359	90%
16-PL-1	25024192	21050268	84%
16-PL-2	31082784	24335041	78%
16-GL-1	24258252	21483296	89%
16-GL-2	23482262	20287636	86%
Mean	26344682	22503935	86%
Total	316136181	270047216	

“-1, -2” means the two biological replicates.
