# Supplementary material for: The Tomato Hoffman’s Anthocyaninless Gene Encodes a bHLH Transcription Factor Involved in Anthocyanin Biosynthesis That Is Developmentally Regulated and Induced by Low Temperatures
Source: PLoS One. 2016 Mar 4;11(3):e0151067. doi: 10.1371/journal.pone.0151067 (PMC4778906; doi:10.1371/journal.pone.0151067)
Supplement: S6 Table — (PDF) [file pone.0151067.s012.pdf]

S6 Table. The enriched GO terms in biological processes of DEGs in 16-PH compared with 16-GH.

	GO term	p-value
Up-regulated genes	phenylpropanoid biosynthetic process	8.15E-04
	cellular amino acid derivative biosynthetic process	0.001859
	aromatic compound biosynthetic process	0.002278
Down-regulated genes	response to carbohydrate stimulus	0.002699
