# Supplementary material for: The Tomato Hoffman’s Anthocyaninless Gene Encodes a bHLH Transcription Factor Involved in Anthocyanin Biosynthesis That Is Developmentally Regulated and Induced by Low Temperatures
Source: PLoS One. 2016 Mar 4;11(3):e0151067. doi: 10.1371/journal.pone.0151067 (PMC4778906; doi:10.1371/journal.pone.0151067)
Supplement: S7 Table — (PDF) [file pone.0151067.s013.pdf]

S7 Table. Primer used in this study.

Primer name	Sequence (5'-3')	
	Forward primer	Reverse primer
For map-based cloning		
CAPS1	CTTAACTTGCCTTAGCCC	CTCCGCAGATAGACCTCA
CAPS2	GCCTCGATGTCCAAAGGTGTAGC	TGGCGTGAGGTTGTCCTGAGTG
CAPS3	GTGAGGATGGGAATAAGGAGAC	ATAAAGAATGCTAACCAGGAGA
CAPS4	AGCGTGAAAGTTTAGGGT	AGCGAGCAGATGGTGATT
CAPS5	CGAAATTATCGGTCCAAA	AACAAGTCCCGTTATCAA
The other molecular markers taken from Tomato-EXPEN 2000 [1] .		
For qPCR		
ACTIN	GGGATGGAGAAGTTTGGTGGTGG	CTTCGACCAAGGGATGGTGTAGC
AH	CTAAGAGTGCCCGCATACAGAC	ATCCGAAGTGGAGTGCTCAGATA
*PAL**	GGAATTGCAGGGTTGCCACTTT	AAGGCCGCGTTGCCTAAAGAAG
*CHS1**	TGGTCACCGTGGAGGAGTATC	GATCGTAGCTGGACCCTCTGC
*AAC**	CCCTCCAGTACCACCAGAAA	TTCAGACAACCTTCCAGCAA
*GST**	TGGGACACAACAGTGATTTGA	TGGCTTAGATCGGCTAAGGA
*5GT**	GTGGCATTTCCTCATTGGAC	TCATCACTCTCAACCACACCA
DFR	GACAGAACGAAGTAATCAAAC	TAAAAGAGAAGAATAGCCAAACG
CHI	GAAGCAGTGCTCGATTCCATAA	GTTTTTCACAAACCAACAGTTCT
F3'5'H	CCCTCAACGCCACTAAATCTCC	TCACTATTCCCATCCTTGTCCCCG
CHS2	GGCCGACTACCAACTCACCAA	GTCCCACCAGCAAAGCAACCTT
*4CL**	GCATTGGAGAATGGTGTGAA	CTCATCGGCCTGAATCAACT
F3H	GTGAAAAGTTGATGGATTTGGC	GTAATGGTTCCTGGATCGGTGTGT
ANS	GAACTAGCACTTGGCGTCGAA	TTGCAAGCCAGGCACCATA
CHI-LIKE	GCGATAGAAGGTAAGGA	AGCCAAAGAAGAAATAGTTGT
RT	CTGGCAATGCAAACAGAGTGA	TCGACTTGCGGAAGAGTGAGA
Solyc06g049020	GGGCGTTTCCTCATCGTGT	CTCGCTGTAAATCTCAACCAACA

*Primer sequences taken from previous study [2].

Reference

1. Fulton TM, Van der Hoeven R, Eannetta NT, Tanksley SD. Identification, analysis, and utilization of conserved ortholog set markers for comparative genomics in higher plants. Plant cell. 2002;14(7):1457-67.
2. Povero G, Gonzali S, Bassolino L, Mazzucato A, Perata P. Transcriptional analysis in high-anthocyanin tomatoes reveals synergistic effect of Aft and atv genes. J PPlant Physiol. 2011;168(3):270-9.
